# Experimental Method for Characterizing Electrical Steel Sheets in the Normal Direction

**DOI:** 10.3390/s101009053

**Published:** 2010-10-08

**Authors:** Nabil Hihat, Jean Philippe Lecointe, Stephane Duchesne, Ewa Napieralska, Thierry Belgrand

**Affiliations:** 1 Univ Lille Nord de France, F-59000 Lille, France; E-Mails: nabil_hihat@ens.univ-artois.fr (N.H.); stephane.duchesne@univ-artois.fr (S.D.); ewa.napieralskajuszczak@univ-artois.fr (E.N.); 2 UArtois, LSEE, F-62400 Béthune, France; 3 ThyssenKrupp Electrical Steel (TKES), F-62330 Isbergues, France; E-Mail: thierry.belgrand@thyssenkrupp.com (T.B.)

**Keywords:** magnetic measurement, permeability, electrical steel sheet, magnetic anisotropy

## Abstract

This paper proposes an experimental method to characterise magnetic laminations in the direction normal to the sheet plane. The principle, which is based on a static excitation to avoid planar eddy currents, is explained and specific test benches are proposed. Measurements of the flux density are made with a sensor moving in and out of an air-gap. A simple analytical model is derived in order to determine the permeability in the normal direction. The experimental results for grain oriented steel sheets are presented and a comparison is provided with values obtained from literature.

## 1. Introduction

Grain oriented **GO** sheets are usually characterised in the directions of the sheet plane only. However, modelling magnetic circuits in 3D, for example using finite element software, often requires the knowledge of characteristics of magnetic materials in all three principal directions. Unfortunately, information about the normal direction, that is to say the direction normal to the sheet plane, is rarely available [1]. Two reasons underline the difficulty. First, it is indeed what happens in the rolling and transverse (which is perpendicular to rolling but still in the sheet plane) directions that is of prime importance regarding magnetic field distribution, whereas the normal flux often generates only secondary effects. Thus, not surprisingly, most studies are focused on the 2D characterisation. But this may prove insufficient in applications such as magnetic cores of power transformers [2]. The amplitude of the normal flux may be small but the surface it crosses is often large, thus resulting in noticeable induced currents, even at low frequency of supply. A typical example is the step-lap joint of a transformer where the effect of such currents may not be negligible [3–5]. Secondly, the measurements to include the characteristics in the normal direction are difficult [6] and very few results have been published. The property of anisotropy change from grain to grain. Some methods of testing the plane distribution of anisotropy are presented in [7,8] A measuring system for 3D magnetic field scanning is presented in [9]. Estimating the influence of eddy currents due to normal flux is a challenge, as explained for example in [10]. The authors estimated the relative permeability *μ_z_* in the normal direction to be close to that of air, whereas results reported in [11–13] seem to suggest that a more realistic value for *μ_z_* is between 100 and 160 for different electrical steel sheets qualities.

The aim of this paper is to present a method for determining the *μ_z_* permeability of a pack of magnetic sheets (insulation and iron) more accurately using static magnetisation. A search coil moving in and out of an air-gap is used and two test benches have been developed. The first is used to explain the principle of the proposed measurement and to point out practical difficulties. An enhanced 3D test bench is then used to determine values of *μ_z_* and compare them with data available from literature for dynamic modes.

## 2. The Principle and the Experimental Device for Static Characterisation

### 2.1. The principle

**Figure 1.**
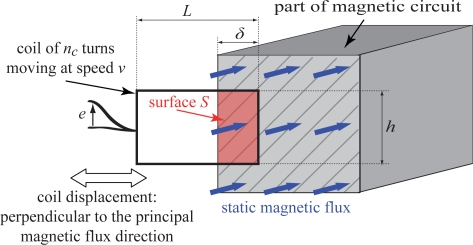
Principle of static excitation.

The principle of the proposed measurement technique is explained with the aid of the first test bench. The originality of the method stems mainly from the fact that the magnetisation is static, that is uses DC excitation. Thus the magnetic flux is constant in time and no eddy currents are induced in laminations, which would otherwise influence the readings. As the flux does not change in time, in order to generate a required electromotive force, the search coil is continuously being moved in and out of the air-gap with sinusoidal velocity *υ*(*t*). The movement is perpendicular to the principal direction of the flux density, shown in Figure 1 as horizontal (right and left). A coil with *n_c_* turns has length *L* and height *h*. The area of the coil which ’cuts’ magnetic flux is *S*(*t*) = *hδ*(*t*) as shown in the Figure 1; as a result an electromotive force (emf) *e*(*t*) is induced. The field distribution in the gap is uniform and thus the flux passing through *S* is *φ*(*t*) = *B* ∫ *d*(*S*(*t*)). The area of coupling between the coil and the magnetic flux changes so that the emf may be expressed as:

(1) $$e(t)=n_{c}\frac{d\varphi (t)}{\mathrm{dt}}=n_{c}B\frac{\mathrm{dS}(t)}{\mathrm{dt}}=n_{c}\mathrm{Bh}\frac{d\delta (t)}{\mathrm{dt}}=n_{c}\mathrm{Bhv}(t)$$

Hence the (constant) flux density *B* depends on the emf peak value *ê*, the coil height *h*, the number of turns *n_c_* and the velocity peak value of the coil movement *υ̂*. *B* is calculated by using Equation 2:

(2) $$B=\frac{\hat{e}}{n_{c}h\hat{v}}$$

### 2.2. The first experimental set up and validation of the method

**Figure 2.**
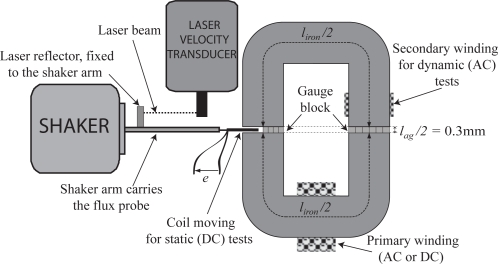
Test bench for static characterization.

The first experimental set up is shown in Figure 2. The magnetic circuit consists of two U-shaped cores, each made of grain oriented laminations of 0.1 mm thickness and having the rolling direction parallel to the vertical part of the U shape (and hence perpendicular in the bottom/horizontal section). The U cores are separated by two 0.3 mm air-gaps. Non-magnetic spacers are used to maintain the separation and the whole assembly is pressed using a bolt. The moving search coil, wound using a 0.05 mm diameter wire, has 5 turns. The coil is moved using a ’shaker’ controlled by a frequency generator. The used shaker (Endevco V406) is an electro-dynamical one: a moving part linked to the structure is attached to a supplied solenoid. In a magnetic field, the moving group is set in motion and, according to current waveform and magnitude, different excitations can be produced: random, pseudo-random, sinusoidal, pulsed. The speed of the movement is measured with a laser velocity meter. The second search coil for dynamic measurements has 150 turns.

The purpose of the first test was to establish hysteresis characteristics for the whole magnetic circuit consisting of the two U-shaped laminations and two air-gaps of Figure 2. Two measurements were taken, one using a DC supply and another at 50 Hz. The proposed method ignores the effects of the leakage flux in the system. In order to justify this simplification, readings were taken to estimate the field around the magnetic circuit, this is discussed in section 3. It was found that flux densities next to the air gap are sufficiently small values to accept the simplification.

The circuital law applied to this simple equivalent magnetic circuit equation yields:

(3) $$H_{c}(l_{\mathrm{iron}}+l_{\mathrm{ag}})=H_{\mathrm{iron}}l_{\mathrm{iron}}+H_{\mathrm{ag}}l_{\mathrm{ag}}=nI$$

where *I* is the supply current, *n* the number of turns in the excitation coil, *H_iron_* the magnetic field strength in the iron, *l_iron_* the length of the ’average’ path for the field in the iron, *H_ag_* the magnetic field in the air-gap (because of symmetry assumed to be the same in both air-gaps), *l_ag_* the combined thickness (length) of the two air gaps and *H_c_* is the equivalent magnetic field in all the magnetic circuit (iron and air gaps). Hence the field in the iron may easily be found as:

(4) $$H_{\mathrm{iron}}(B)=\frac{\mathrm{nI}-H_{\mathrm{ag}}l_{\mathrm{ag}}}{l_{\mathrm{iron}}}=\frac{\mathrm{nI}}{l_{\mathrm{iron}}}-\frac{{\mathrm{Bl}}_{\mathrm{ag}}}{\mu_{0}l_{\mathrm{iron}}}$$

where the equality *B_ag_* = *B_iron_* = *B* of the magnetic flux density in the air gaps and the iron is assumed by the conservation of the normal magnetic flux density component. It should be noted that the expression relies on subtracting two values which may be close to each other in comparison to the length of the air gaps which must be minimized, to avoid reducing the method accuracy.

**Figure 3.**
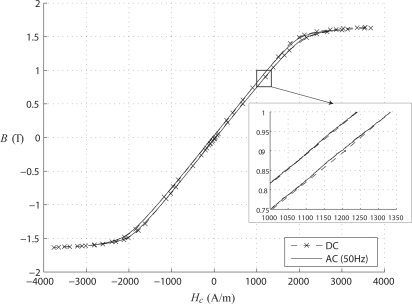
Hysteresis cycle of the whole circuit.

Figure 3 shows hysteresis loops for the complete circuit measured at 50 Hz and using static excitation; the two curves are practically identical with differences of cycle areas not exceeding 9%. The slight differences may be attributed to the influence of eddy currents induced and leakage inductance under AC conditions; however, the effect is very weak and thus the static measurement may be considered as appropriate.

**Figure 4.**
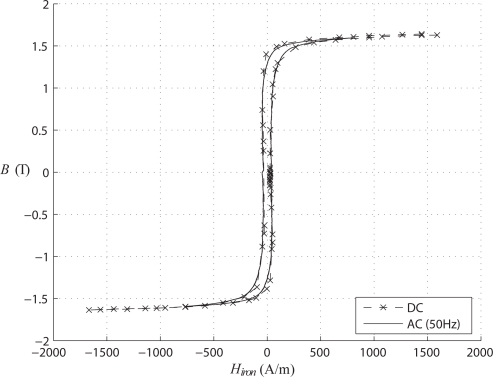
Hysteresis cycle of the iron (U core).

Another comparison is presented in Figure 4, where the iron hysteresis loops are computed, for dynamic (at 50 Hz) and static conditions, by using Equation 4. In comparison with the overall hysteresis loop, one can conclude that most of the energy is consumed in the air gaps. Again, the two curves practically coincide thus validating the principle of the proposed method.

The first experimental rig described above has served the purpose of verifying the principle of the proposed method but it has limited applicability for practical measurements. The main limitation is the maximum depth of the air-gap of 0.3 mm thus allowing only up to one lamination to be tested at a time, with regards to the average length of iron (on the order of tens centimeters), potentially leading to inaccuracies. That is why a second rig has therefore been designed and built.

## 3. An Enhanced Experimental Rig for Measuring Normal Permeability

### 3.1. Description

**Figure 5.**
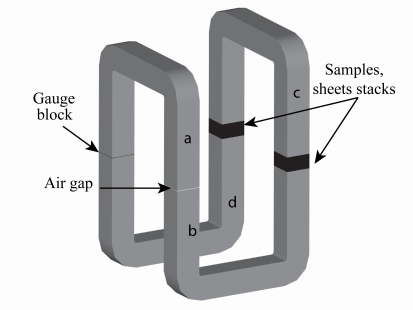
Test bench for static characterisation.

An enhanced experimental rig for practical measurements of normal permeability is depicted in Figure 5. It has a more complicated design consisting of four U shaped pieces and four air-gaps, two of which have fixed depth of 0.6 mm. The search coil is moved in and out of one of them, placed between pieces *a* and *b*. The air-gap between *a* and *d* is filled with a non-magnetic spacer (a ’gauge block’) to maintain symmetry. The remaining two air-gaps, between *c* and *b* and between *c* and *d*, have adjustable thickness but varied simultaneously, again to preserve symmetry. These two gaps are used to accommodate magnetic samples which have the same area as the cross section of the U core (50 mm × 50 mm); the number of laminations should be the same in both air-gaps and there is really no restriction on the number of sheets in a sample.

### 3.2. Field uniformity and leakage

**Figure 6.**
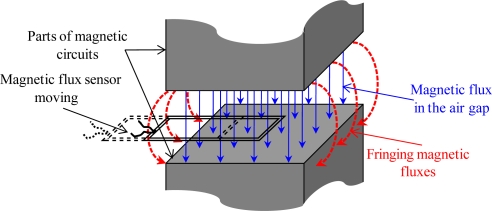
Fringing magnetic flux.

**Figure 7.**
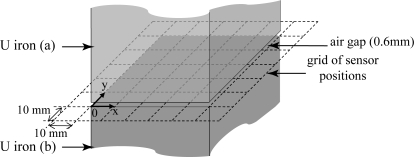
Sensor positions in the airgap plane.

The main sources of potential inaccuracies of the proposed method are lack of uniformity of the magnetic field distribution in the air-gap and existence of fringing (leakage) fluxes, as such leakage is neglected in calculations. A simple diagram in Figure 6 explains the fringing flux problem. To minimise these effects the excitation winding has been designed to consist of four sections, each of 300 turns, distributed uniformly along the magnetic circuit, with the exception of the corners. Several measurements have been taken in an attempt to provide some quantitative assessment of the problem. A search coil of 5 turns and dimensions 10 mm × 10 mm is placed in the airgap at positions defined by a regular ’grid’, as shown in Figure 7. Such dimensions have been imposed by chosen for 2 reasons. The first reason concerns the feasibility of the sensor, which thickness does not exceed 0,2 mm. The second reason concerns the amplitude of the emf. No exploitable signal can be measured with accuracy if the surface of the sensor is too small.

**Figure 8.**
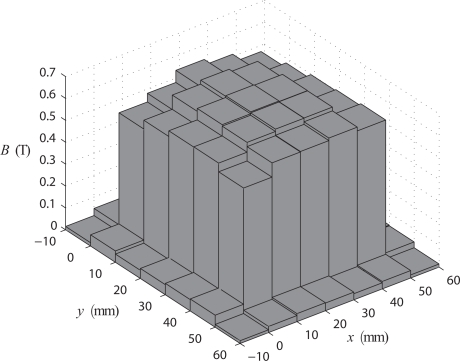
Magnetic flux density distribution in the airgap plane.

A five turns secondary winding is placed on the U iron *a* to measure the average flux density. The measured flux density distribution inside the air-gap and 1 cm away from its edge is shown in Figure 8, for a value of magnetic flux density measured with the secondary winding of 0.65 T at 1 Hz. The average magnetic flux density of the 25 elements (*x* ∈ [0, 50] and *y* ∈ [0, 50]) in the air gap is about 0.64 T. The difference against the magnetic flux density measured with the secondary winding (0.65 T) is only about 1.2%. Under the air gap, the local flux densities values, in comparison with the global magnetic flux, are varying from −6.5% to +5%, especially close to the edges. This is partly due to the U cores cross sections which have 1 mm tolerance. The geometry tolerance of the U cores cross sections represents 10% of the search coil area. This provides some idea about the practical field uniformity. What matters more, however, is not so much the local distribution but the effect of the non-uniformity on the total flux. Regarding to the magnetic flux densities measured around the air gap, its maximum value is only about 0.05 T which is most satisfying and increasing confidence in the proposed approach.

### 3.3. Static and dynamic measurements

**Figure 9.**
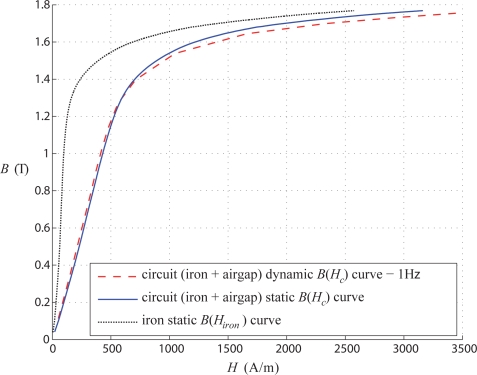
Static and dynamic characterizations of the 3D-cores.

Figure 9 shows measured static and dynamic (at 1 Hz) magnetic characteristics for the complete magnetic circuit without any samples (with air-gaps, between *c* and *b* and between *c* and *d*, reduced to zero), and additionally a computed curve for the iron part of the circuit using 3. The two measured curves for the full circuit are almost identical, the differences do not exceed 4.5%. The computed curve for the iron path only is clearly different and will allow the estimation of the characteristics of the samples in the normal direction as explained in the next section.

## 4. Results

### 4.1. Magnetic permeability in the direction normal to lamination plane

Tests were conducted using 100 grain oriented laminations (50 in each of the two air gaps) of 0.35 mm thickness (M140-35S) supplied by *ThyssenKrupp Electrical Steel Company*; the power losses at 1.7 T at 50 Hz is 1.40 W/kg [14]. Measurements were first taken without samples, and then with laminations in position, for the same geometry (the same size of the air-gaps). The excitation coil is also wounded around the samples in order to limit the flux leakage and the flux fringing. Then, the magnetic permeability in the direction normal to the lamination plane has been calculated using the relationship:

(5) $$\mu_{z}(B)=\frac{n_{s}l_{s}B}{\mu_{0}\left(\mathrm{nI}-H_{\mathrm{iron}}(B)l_{\mathrm{iron}}-\frac{B}{\mu_{0}}l_{\mathrm{ag}}\right)}$$

where *n_s_* is the number of samples and *l_s_* lamination thickness (0.35 mm in this case); the expression in the denominator ( $H_{\mathrm{iron}}(B)l_{\mathrm{iron}}+\frac{B}{\mu_{0}}l_{\mathrm{ag}}$) is known from 3 and refers to the ampere-turns absorbed in the iron and air-gaps. Therefore, knowing with accuracy *l_iron_* and *l_ag_* is not required to determine *μ_z_*. The value is given, point by point for the measured value of *B*, by the curve *B*(*nI*) obtained without the samples. The permeability of the iron *μ_z,iron_* can also be determined with the following expression:

(6) $$\mu_{z,\mathrm{iron}}=\frac{n_{s}(l_{s}-2l_{i})B}{\mu_{0}\left(nI-H_{\mathrm{iron}}(B)l_{\mathrm{iron}}-\frac{B}{\mu_{0}}(l_{\mathrm{ag}}+2n_{s}l_{i})\right)}$$

where *l_i_* is the sheet insulation thickness on one face.

**Figure 10.**
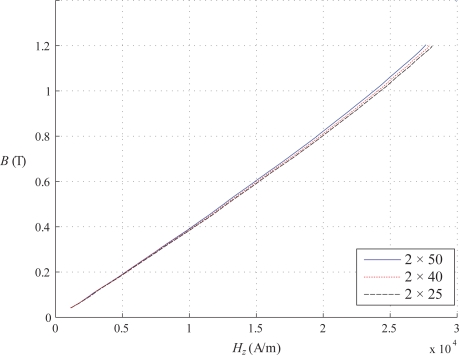
Normal permeability *μ_z_* of the sheet samples for various *n_s_*.

The Figure 10 presents the results for 3 different sample numbers: 2 × 25, 2 × 40 and 2 × 50. It shows that *μ_z_* has same values whatever the number of sheets. The pressure over the two packs of sheets is constant: it is due to the own weight of the U-core over them. A series of ten tests has been undertaken under the same conditions, which allowed for the average characteristic to be computed as presented in Figure 10. More tests were done but results were practically the same, hence repeatability of measurements was assured.

**Figure 11.**
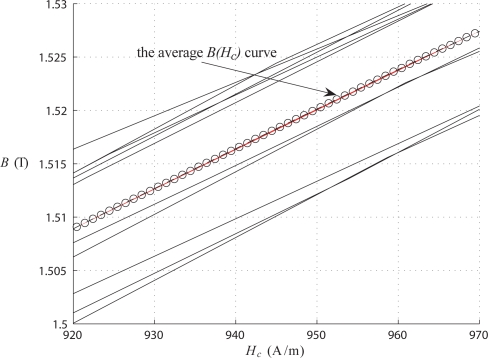
Measurement dispersion of the curve *B*(*H_c_*).

To provide further details, an enlarged small portion (’zoomed in’) of the measured characteristics is shown in Figure 11 with all ten curves drawn; for example, at the magnetic field strength of 945 A/m the variation of the measured value of flux density is from 1.5 T to 1.525 T, thus within 1%. The magnetic characteristic *B*(*H_z_*) (with *H_z_* = *B*/*μ*_0_*μ_z_*(*B*)) computed on the basis of these measurements is practically linear. The relative permeability in the normal direction has been found to be between 28.6 and 34.2. This finding may seem surprising, or even shocking, but has some support in literature [12,13]. It is believed that the main reason for inaccuracies in previously reported results could have been related to the significant, but neglected, effect of eddy currents induced by the normal flux; such currents have been virtually eliminated in our proposed measuring technique thanks to the use of static excitation.

### 4.2. Error analysis

We can report repeatability of the measurements for different number of laminations and various types of electrical steel sheets. This paper only presents results for the linear part of the magnetic characteristic *B*(*H_z_*) (flux densities up to 1.2 T). The effects of saturation will be subject of a separate publication. Measurement errors are introduced by several components of the experimental rig and can be estimated as follows:

- measurement of *I* and *e* may be subject respectively to errors up to 1.8% and 2.3%; the measured range varies from 30 mA to 8 A,
- the total harmonic distortion (THD) of the velocity *υ*(*t*) signal, implying the same THD for the emf *e*, is only about 0.5% and its measurement accuracy is 0.2%,
- the width of the moving search coil has been taken as *h* = 35 mm, the accuracy of this measurement is assumed to be 0.5 mm.

Overall, the accuracy of the entire measuring technique of *μ_z_* is estimated to be 7.5%. At last, let us point out that value of *μ_z_* results from the averaging of several measurements.

The authors point out effect of demagnetizing field $H_{z}^{d}$ [15]. Indeed, each sample stack constitutes a magnetic dipole in which *H_z_* results from the magnetizing field $H_{z}^{m}$ and from the demagnetizing field $H_{z}^{d}$, as expressed in 7.

(7) $$H_{z}=H_{z}^{m}+H_{z}^{d}$$

Therefore, because of the difficulty to separating $H_{z}^{d}$ from *H_z_*, the magnetic characteristics in the normal direction is expressed, as commonly in the literature [12], with *H_z_*.

## 5. Conclusions

The proposed technique for measuring magnetic characteristics in the direction normal to the lamination plane is claimed to be more reliable as it eliminates the influence of planar eddy currents. This is achieved by using static excitation and a moving search coil, an arrangement which we consider original and not reported before.

It has commonly been assumed—as indeed found frequently in literature—that the magnetic characteristic in the normal direction (often referred to as *z*) is similar to the perpendicular direction (*y*, taken as perpendicular to the direction of rolling for grain oriented sheets); indeed, some commercial field simulation programs automatically make such substitution, for both linear and non-linear regimes. Our results appear to suggest that the characteristics in the normal direction are in fact significantly different. Moreover, for the unsaturated part of the curve, the relative normal permeability remains reasonably constant and is in the range between 42 and 54 for high quality laminations used in our experiments. We expect that using these values will have a direct effect on the modelling results, especially in 3D simulations. In the matter of the demagnetization phenomena, the determination of the demagnetizing field is not simple because of the dimensionless of the demagnetization coefficient.

Our future work will expand the testing programme to include other materials, both isotropic and grain oriented, further study the effects of saturation and finally consider the possible influence of the inter-lamination insulation. We also envisage incorporating the normal permeability into our 3D modelling.
